# Progress in Medicine

**Published:** 1897-10-30

**Authors:** 


### PROGRESS IN MEDICINE.

### OBSTETRICS.

**Abortion.**—Skutsch,[1] of Jena, has lately described a case of tubal pregnancy in which the ovum was discharged through the uterus and vagina. Cases of this nature have been often described, but the possibility of this termination of a tubal pregnancy is remote, while an error in diagnosis is very probable. Tubal abortion proper—*i.e.*, the expulsion of the ovum into the peritoneal cavity—is a well-known phenomenon, explicable by the occurrence of fertilisation and arrest of the ovum close to the outer extremity of the Fallopian tube, and the dilatation of the free extremity until the ovum can pass through it. The passage into the uterus, however, through the smaller isthmus and uterine opening, seems most unlikely, and the implantation of the fertilized ovum in this part of the tube is well known to lead to early rupture. Skutsch maintains that the uterine wall may rupture on that side which projects into the uterine cavity, with discharge of the ovum through the uterine cavity and vagina. He describes a case where the discharge of a thick and complete decidua was followed by that of an ovum which could never have lain in the decidual cavity. He seems to consider that there was no possibility of bicornuate uterus or similar malformation being present.

A case recorded by so careful an observer demands consideration, but many sources of fallacy must be excluded before accepting such a course of events as usual or even likely. The causation of abortion and premature labour has been studied by Romheld[2]; he attributes of the total number (225 cases) studied by him to syphilis, 27 per cent.; retroflexion, 18 per cent.; chronic metritis and endometritis, 10 to 15 per cent.; uterine fibroids, 4·7 per cent.; abnormalities of placenta, 4 per cent.: anteflexion of uterus, 3·5 per cent.; molar pregnancy, 1 per cent.; Bright's disease and lateral deviations of the uterus, 5 per cent.

Charpentier[3] adds, as a local cause, infantile uterus, with which is associated mal development and irritability of the muscular coat of the uterus. He insists that in flexions the space at the angle of flexion hypertrophies and interferes with uterine development. He also alludes to congestion of the body and cervix and endometritis, and recommends curetting of the cervical canal even in pregnancy, as after this operation cases which have previously aborted go on to full term. Lefour considers that the use of the intra-uterine stem by causing the uterus to become tolerant of bodies within its cavities will often lead eventually to normal pregnancy.

**Missed Abortion.**—Three cases are described by Kossier[4] in which the ovum was retained in utero for periods of six weeks, two months, and four months, respectively; in two cases, after amenorrhœa of some weeks' duration, hæmorrhage occurred, with symptoms of abortion; no ovum was discharged, and slight, irregular hæmorrhage persisted, which led to the emptying artificially of the enlarged uterus; in each case an ovum was removed, more or less altered, the fœtus of which had been dead for some weeks. In the third case, a sudden hæmorrhage followed six months' amenorrhœa; an ovum was removed containing a three months' fœtus which had died at that period, but the discharge of which had been delayed three months.

Two papers on the pathology of this condition, by Budin and Breus, are discussed by W. E. Fothergill,[5] in the first of which the discharge of the ovum occurred on artificial stimulation eleven months after her last normal menstruation, and seven months after the attack of pain and hæmorrhage which seemed to suggest the attempt at natural abortion.

The mass expelled weighed 300 grammes, and measured $3\frac{1}{2}$ by $2\frac{1}{2}$ inches. From without inwards it consisted of old fibrinous adherent clots, which formed two-thirds of the whole mass; within this an altered ovum containing no fœtus, cord, or liquor amni. There were thirteen tumours projecting into the cavity, varying in size from that of a nut to that of a pigeon's egg, dark purple in colour, pedunculated and consisting of slightly-altered blood clot, covered by amnion alone. Budin ascribes their origin to hæmorrhage from the decidua, the blood having passed through ruptures in the chorion to form sub-amniotic hæmatomata.

Breus[6] describes five cases which supplied the material he examined; the shortest term of amenorrhœa was five months, the longest fifteen. Three ova were of the size of a fist, the other two smaller; all contained embryos and a few drops of liquor amni. The cavity was practically filled by excrescences covered with amnion and chorion, which bulged into it. The connective tissue layer of the amnion and chorion was continuous; there was no trace of blood-vessels, and the chorionic villi were absolutely non-vascular; the chorionic epithelium was well preserved, and in many positions showed epithelial beads. The decidua was very vascular, with numerous spindle and small round cells.

The embryos were all of about the second month. The tumours which projected into the cavity owed their origin not to primary hæmorrhage, but to hæmorrhage occurring into pouchings and folds of the coverings of the embryo previously formed. There is no proof that the pouchings occur before the hæmorrhage, nor, as stated by Breus, that the ovular structures grow after the death of the fœtus. These cases have considerable pathological interest in connection with malignant deciduoma, and also from a medico-legal point of view.

**Ectopic Gestation.**—Several cases of this affection have been reported lately, and many interesting points raised. G. H. Balleray[7] mentions some points in the diagnosis and treatment of this affection in its early stages, and described a case in which, after irregular menstruation for nine months, colicky pains in the abdomen, requiring the injection of morphia, came on. The uterus was found to be pushed to the right by an elastic and immovable mass on the left side, in the posterior half of the pelvis. Tubal pregnancy was diagnosed; on abdominal section a fœtus was found in each tube, that on the left being the larger; drainage was used, and this prevented the shock which supervened on the operation being diagnosed as collapse from hæmorrhage and leading to the opening up of the abdomen. The diagnosis was made solely on physical signs. In another case the sac ruptured whilst the patient was being removed to hospital, about a quart of clots and blood being removed from the abdomen at the operation.

The diagnostic points appear to be cessation or irregularity of menses, followed, after a time, by pain, often colicky, and discharge of blood per vaginam, with a mass in the pelvis on one side or behind the uterus, the symptoms of pregnancy which are present being found not to have any connection with a normal pregnancy.

The frequency with which rupture of the sac takes place within a month of the commencement of the tubal pregnancy is discussed at length by Hastings Gilford,[8] and his arguments based on two cases. In the first, the symptoms pointed to rupture of the tube, and a swelling was found at the side of the uterus, which disappeared after some time; no operation was performed. The second case was particularly interesting, in that the symptoms all pointed to rupture of a gastric ulcer, the pain being referred to the region of the sternum and shoulders, and associated with vomiting and retching, while internal hæmorrhage was evidently going on. The abdominal tenderness also, which was uniform, was more marked in the epigastric region. There was dulness in the left flank, and bimanual examination failed to give any information. The left tube was found to be ruptured, and the ovary contained a recent corpus luteum. In both these cases menstruation had occurred about a fortnight before the symptoms which led to the suspicion of rupture in the first case, and to operation in the second.

[1] B. M. J., July 10, 1897. [2] Centralblatt für Gynak, quoted in N. Y. Med. Record, April 17, 1897. [3] Ep. B. M. J., July 10, 1897. [4] Rev. Med. de la Suisse Rom., quoted Ep. B. M. J., May 15, 1897. [5] Med. Chron., Feb., 1897. [6] Med. Chron., loc. cit. [7] Med. Record, April 13, 1897. [8] Lancet, Feb. 20, 1897.

